# Carnosic Acid Protects INS-1 β-Cells against Streptozotocin-Induced Damage by Inhibiting Apoptosis and Improving Insulin Secretion and Glucose Uptake

**DOI:** 10.3390/molecules27072102

**Published:** 2022-03-24

**Authors:** Waseem El-Huneidi, Shabana Anjum, Mohamed A. Saleh, Yasser Bustanji, Eman Abu-Gharbieh, Jalal Taneera

**Affiliations:** 1 Department of Basic Medical Sciences, College of Medicine, University of Sharjah, Sharjah 27272, United Arab Emirates; ybustanji@sharjah.ac.ae; 2 Sharjah Institute for Medical Research, University of Sharjah, Sharjah 27272, United Arab Emirates; anjum6repro@gmail.com (S.A.); mohamed.saleh@sharjah.ac.ae (M.A.S.); eabugharbieh@sharjah.ac.ae (E.A.-G.); 3 Department of Clinical Sciences, College of Medicine, University of Sharjah, Sharjah 27272, United Arab Emirates; 4 Department of Pharmacology and Toxicology, Faculty of Pharmacy, Mansoura University, Mansoura 35516, Egypt; 5 Department of Biopharmaceutics and Clinical Pharmacy, School of Pharmacy, The University of Jordan, Amman 11942, Jordan

**Keywords:** carnosic acid, streptozotocin, apoptosis, pancreatic β-cells, diabetes, insulin secretion, INS-1 cells

## Abstract

Carnosic acid (CA), a natural polyphenolic diterpene derived from *Rosmarinus officinalis*, has been proven to possess a broad spectrum of medicinal properties. Nevertheless, no studies on its impact on pancreatic β-cells have been conducted to date. Herein, clonal rat INS-1 (832/13) cells were pretreated with CA for 24 h and then incubated with streptozotocin (STZ) for 3 h. Several functional experiments were performed to determine the effect of CA on STZ-induced pancreatic β-cell damage, including cell viability assay, apoptosis analysis, and measurement of the level of insulin secretion, glucose uptake, malondialdehyde (MDA), reactive oxygen species (ROS), and proteins expression. STZ treatment decreased cell survival, insulin secretion, glucose uptake, and increased apoptosis, MDA, and ROS production in INS-1 cells. Furthermore, protein expression/phosphorylation analysis showed significant down-regulation in insulin, PDX-1, PI3K, AKT/p-AKT, and Bcl_2_. On the other hand, expression of BAX and BAD and cleaved PARP were significantly increased. Interestingly, preincubation with CA reversed the adverse impact of STZ at the cellular and protein expression levels. In conclusion, the data indicate that CA protects β-cells against STZ-induced damage, presumably through its modulatory effect on the different pathways, including the Pi3K/AKT/PDX-1/insulin pathway and mitochondria-mediated apoptosis.

## 1. Introduction

Diabetes is a long-term metabolic disorder characterized by chronically high blood glucose levels [1]. According to the World Health Organization (WHO), the prevalence of diabetes exceeds 8% globally, and by 2030, it is anticipated to be the seventh leading cause of death [2]. Type 1 (T1D) and type 2 diabetes (T2D) are the two most common types of diabetes. T1D is defined by insulin shortage caused by autoimmune β-cell destruction, whereas a gradual decrease in insulin production and/or impaired insulin action is the main characteristic of T2D [3]. Typically, long-term hyperglycemia is associated with serious consequences in the kidneys, nerves, heart, eyes, and lower extremities [4].

Dysfunctional pancreatic β-cell is the hallmark in the pathogenesis of both types of diabetes. While T1D is linked with significant loss of pancreatic β-cells [5], several reports indicated a marked reduction in pancreatic β-cell mass in T2D [6,7]. Additionally, prolonged hyperglycemic conditions may also increase glycolysis in β-cells and subsequent ROS production via multiple routes [8]. High levels of ROS induce oxidative stress, which leads to pancreatic cell damage and apoptosis [8]. Hence, factors such as β-cell proliferation, apoptosis, regeneration, and ROS levels play an essential role in the progression of diabetes [9,10]. Therefore, preserving and restoring β-cell mass and function during early-diagnosed patients with diabetes is crucial for beneficial diabetes therapy [10].

**Figure 1:**
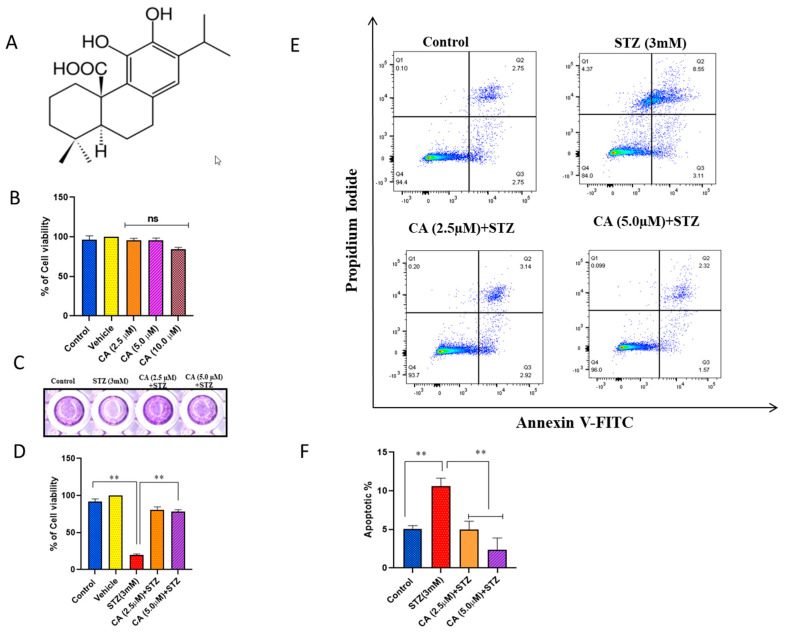
Impact of CA or STZ treatment on cell viability and apoptosis in INS-1 cells. (**A**) CA chemical structure. (**B**) MTT assay in INS-1 cells preincubated with various concentrations of CA (2.5, 5, 10 µM) for 24 h compared to untreated cells. (**C**) Crystal violet staining for INS-1 cells. (**D**) MTT assay in STZ-treated cells (3 mM) or preincubated cells with 2.5 or 5 µM of CA for 24 h followed by STZ-treated versus untreated control cells. (**E**) Annexin V-PI analysis of apoptosis in untreated control cells, STZ-treated cells for 3 h, or cells pretreated with CA for 24 h prior to STZ exposure. Q1, Q2, Q3, and Q4 represent the necrotic, late apoptotic, early apoptotic, and viable cell populations. (**F**) Apoptotic index summarizes the apoptosis results. Data were obtained from three independent experiments. ** *p* < 0.01. Bars represent mean ± SD.

Bioactive compounds isolated from natural sources, primarily herbal plants, have exhibited various pharmacological activities. In this context, CA, shown in Figure 1A, is a polyphenolic abietane diterpene, found naturally in plants such as *Rosmarinus officinalis*, and has been linked to multiple pharmacological properties, comprising anticancer, anti-inflammatory, and antiviral activities [11,12,13]. Previous studies have reported a significant improvement in glucose tolerance, reduction in fasting blood glucose, and enhancement of insulin sensitivity through decreasing ROS accumulation upon treating diabetic mice with CA [14,15]. Other lines of evidence have found that CA improved glomerular sclerosis, mesangial expansion, and protected against diabetic nephropathy primarily via the Nrf2/NF-B pathway [16]. 

However, the potential protective impact of CA in β-cell damage is still unclear. Thereby, this work aimed to investigate the potential effect of CA on STZ-induced damage in rat insulinoma (INS-1 832/13) cells and explore the signaling pathway underlying the mechanism of action. 

## 2. Results

### 2.1. CA Reverts the Cytotoxic Effect of STZ Treatment in INS-1 Cells 

The cytotoxic effect of CA on INS1 cells was tested using the MTT cell viability assay in cells treated with different concentrations (2.5, 5 and 10 µM). As shown in Figure 1B, the results revealed that CA treatment has no cytotoxic effect on INS-1 cells compared to untreated cells. On the other hand, as expected, treating INS-1 with STZ (3 mM) for 3 h led to a significant reduction in cells viability (80%; *p* < 0.05) (Figure 1C,D). However, INS-1 cells, preincubated with 2.5 or 5 µM of CA, significantly reduced (*p* < 0.05) the cytotoxic effect of STZ as illustrated in Figure 1B. STZ treatments showed an apoptotic effect on INS-1 cells as measured by Annexin V-PI staining (Figure 1E). The total number of apoptotic cells and necrotic cells were significantly increased (*p* < 0.05) in STZ-treated cells compared with untreated (Figure 1E, upper panels). Similarly, CA exhibited a protective effect by reversing the apoptotic and necrotic cells to normal after STZ treatment, as shown in Figure 1E,F.

### 2.2. CA Enhances the Insulin Secretion in STZ-Treated INS-1 Cells

**Figure 2:**
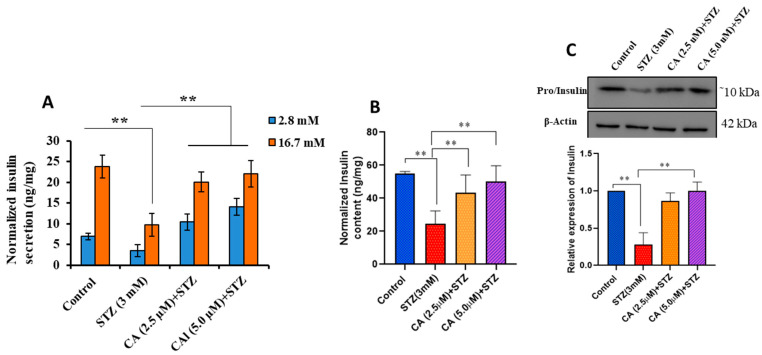
Impact of CA on insulin secretion, content, insulin expression in INS-1 cell treated with 3 mM STZ treatment. (**A**) Normalized stimulated insulin secretion was determined by ELISA in response to 2.8 and 16.7 mM glucose, respectively. (**B**) Normalized insulin content. (**C**) Western blot analysis of INS (Pro/Insulin), all in STZ-treated cells or cells preincubated for 24 h with CA followed by STZ treatment compared to control cells. β-actin was used as a reference control for Western blot. Data presented are obtained from three independent experiments. ** *p* < 0.01, and bars represent mean ± SD.

As shown in Figure 2A, INS-1 treated with STZ exhibited a significant reduction in glucose-stimulated insulin secretion (GSIS) at both basal (2.8 mM) and stimulation (16.7 mM) glucose concentrations (~45, respectively, %; *p* < 0.01) compared with untreated cells. Interestingly, cells preincubated with 2.5 or 5 µM of CA followed by STZ treatment had better insulin secretion capability than STZ-treated cells alone (Figure 2A). Furthermore, the improvement in insulin secretion in preincubated CA cells was also associated with a significant rise in insulin content at 2.5 or 5 µM (~50%; *p* < 0.01) (Figure 2B). To further confirm these data, we analyzed the protein expression of Pro/Insulin. Western blot analysis revealed a significant reduction in Pro/Insulin in the STZ-treated cells compared with control cells. In contrast, CA preincubated cells restored Pro/Insulin expression (Figure 2C).

### 2.3. CA Decreases Intracellular ROS and MDA Levels and Increases Glucose Uptake in STZ-Treated INS-1 Cells

**Figure 3:**
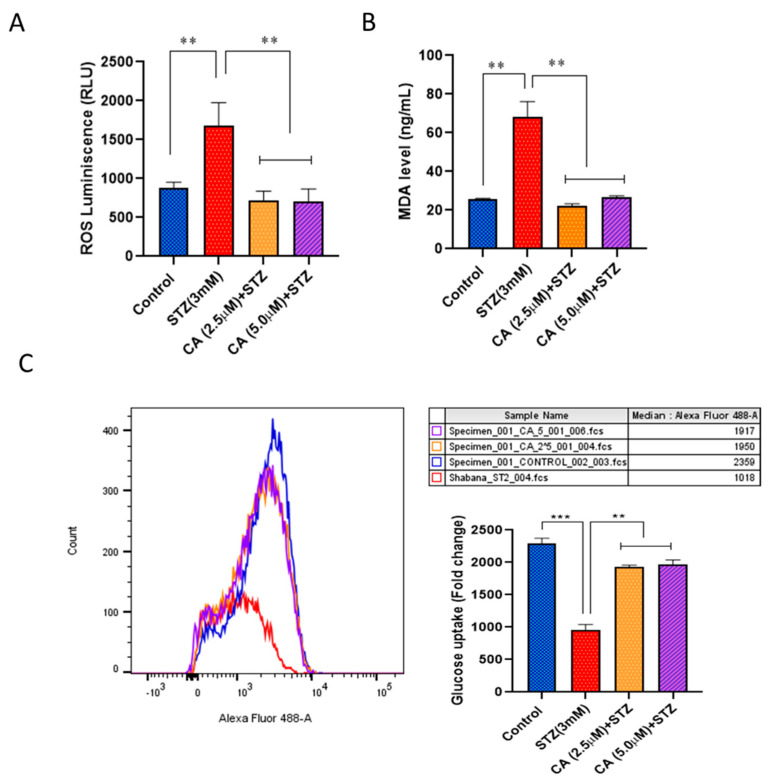
Impact of CA on (**A**) ROS production, (**B**) MDA levels, and (**C**) glucose uptake in STZ-treated cells. Levels of ROS and MDA were determined by fluorescence intensity, while glucose uptake assessment was measured by flow cytometry. Data presented are obtained from three independent experiments. ** *p* < 0.01, *** *p* < 0.001. Bars represent mean ± SD.

As illustrated in Figure 3A,B, ROS luminescence (RLU) and MDA measurements levels in STZ-treated cells revealed a significant elevation (*p* < 0.01) compared with untreated control cells. Preincubation with CA at both doses significantly reduced ROS luminescence (RLU) and MDA levels (*p* < 0.01) when compared with STZ treated cells. Moreover, glucose uptake was reduced considerably in STZ-treated cells (60%; *p* < 0.01) compared with control cells (Figure 3C). Interestingly, CA at both doses reverted glucose uptake activity, similar to average levels in untreated cells (Figure 3C).

### 2.4. Impact of CA on the Expression of Key Proteins in Insulin Production Pathways and the Mitochondria-Mediated Apoptosis Pathway

**Figure 4:**
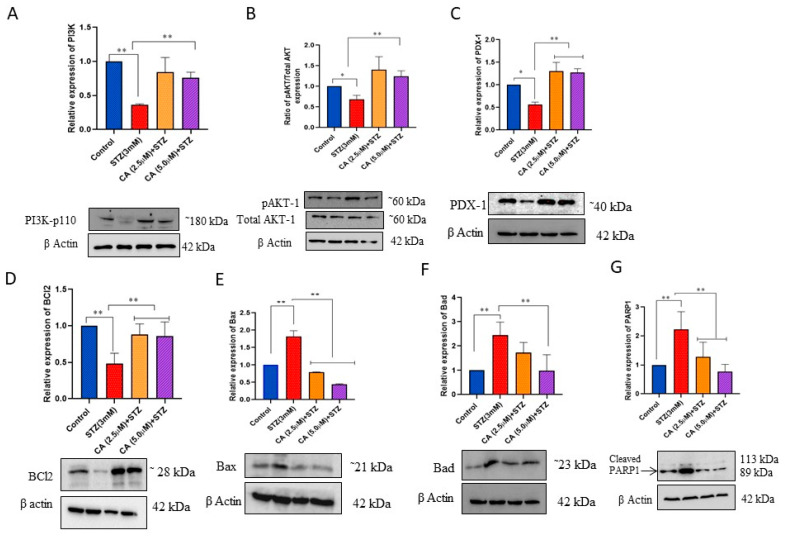
Effect of CA on protein expression. Western blot analysis of PI3K (**A**), AKT/p-AKT (**B**), PDX1 (**C**), BCL2 (**D**), BAX (**E**), BAD (**F**), and cleaved PARP1 (**G**) in STZ-treated cells for 3 h or cells preincubated with CA for 24 h followed by STZ treatment, compared to control cells. β-actin was used as an internal control. Data presented are obtained from three independent experiments. * *p* < 0.05 and ** *p* < 0.01. Bars represent mean ± SD.

To shed more light on the potential molecular biological mechanism underlying the effect of CA, we investigated the expression of key proteins related to Pi3k/AKT/PDX-1 signaling pathway in STZ treated cells with or without CA preincubation using Western blot. As shown in Figure 4, cells treated with STZ showed a significant (*p* < 0.05) decrease in expression of PI3K (Figure 4A), AKT/p-AKT ratio (Figure 4B), and PDX-1 (Figure 4C). This effect was reversed upon the preincubation with 2.5 or 5 µM of CA, as shown in Figure 4. Furthermore, to investigate the potential role of BCl-2 family members in the apoptosis observed with STZ treatment, the expression of anti-apoptotic protein BCL_2_ (Figure 4D) was decreased in STZ treated cells. In contrast, the expression of proapoptotic proteins Bax and Bad (Figure 4E,F) and apoptotic signaling molecules (cleaved PARP) (Figure 4G) were increased, indicating that STZ induced mitochondria-mediated apoptosis. This effect was reversed upon treating the cells with 2.5 and 5 µM CA. 

## 3. Discussion

Hyperglycemia, inflammation, oxidative stress, ROS, and MDA production are often associated with the development and progression of diabetes [17]. The current diabetes drugs intention to alleviate the underlying pathological processes, including lifestyle and weight management and medication/drug interventions [18]. Nowadays, there are several commercial diabetes therapies; however, there is a continuous need to search for more effective new drugs with fewer side effects and lower costs. Several modern drugs are originated from plant sources, including aspirin, digoxin, quinine, and morphine [19]. In this context, herbs with antidiabetic effects are still prevalent; for example, Metformin, the first-line treatment for T2D management, is purified from the French lilac *Galega officinalis* L. [20]. 

The current study demonstrated that CA has no cytotoxic effect on pancreatic β-cells within the tested concentrations. Importantly, we showed that CA protects β-cell against STZ-induced apoptosis, enhances insulin secretion/content and glucose uptake, and decreases ROS and MDA production. Interestingly, preincubation with CA reversed the adverse impact of STZ at the molecular level through increasing the expression/phosphorylation of insulin, PDX-1, PI3K, AKT/p-AKT, and BCL2. At the same time, Bax, Bad, and cleaved PARP expression significantly decreased. Up to our knowledge, this is the first report to look into the role of CA specifically on pancreatic β-cells. Our data align with other findings showing CA’s antioxidative and antiglycative effect by reducing the formation of malondialdehyde and advanced glycation end-products [21,22].

CA is a polyphenolic found naturally in rosemary extract [23]. The phenolic content level varies based on the plant’s anatomical structures, whereby the highest concentration of polyphenols is found in the leaves [19]. CA has been reported to have antioxidant properties that protect against ROS-induced damage [21,24]. Several reports evaluated the potential antidiabetic effect of CA [25]. Wang et al. reported that CA exhibits the antidiabetic action by inhibiting amylase and glucosidase enzymes [21]. Moreover, diabetic rats treated with CA elicited a reduction in blood glucose and were protected against oxidative damage in the liver, kidney, and heart [22]. Interestingly, CA had a prebiotic effect on the gut microbiota, as evidenced by an increase in the diabetes-resistant bacterial population and a decrease in the diabetes-sensitive bacterial population [22]. Mice fed with an HF diet and on CA supplementation showed decreased bodyweight, glucose levels, and insulin levels compared with control mice [26], indicating that CA has anti-obesity properties.

In our experimental setup, STZ was used to induce pancreatic β-cells destruction. Typically, STZ is a glucose analogue that is transported into the β-cells via the GLUT2 transporter [27]. STZ can cause pancreatic cell death by several mechanisms, including nitric oxide (NO) overproduction, activation of DNA repair systems, DNA methylation, and free radical generation, as well as apoptosis and ROS production [28]. 

PI3K/AKT signaling pathway is crucial in regulating cellular activities, such as cell growth, glucose homeostasis, protein biosynthesis, lipid metabolism, and cell survival [29]. In addition, it is well established that PI3K/Akt signaling plays an important role in regulating the function of β-cells especially through activating PDX1, a transcriptional factor that induces insulin production [30,31,32]. Inactivation of PI3K/Akt in β-cells has decreased insulin secretion and glucose intolerance through downregulating PDX-1 [31]. In this context, we found that PI3K/Akt/PDX-1 signaling was considerably reduced in STZ-treated INS-1 cells. In contrast, preincubation with CA enhanced Akt phosphorylation and subsequently increased PDX-1 expression leading to enhance insulin expression and secretion.

The important role of BCL-2 family members in regulating apoptosis has been elucidated in the literature [33]. It is well known that an anti-apoptotic member of the BCL-2 family (such as BCL2 protein) is down-regulated in mitochondria-mediated apoptosis while the proapoptotic members of the BCL-2 family (such as BAX and BAD), in addition to apoptotic signaling molecules (such as cleaved PARP), are upregulated in apoptosis [33]. Our findings are consistent with this mechanism where STZ treatment induces apoptosis apparently through mitochondria-mediated pathways through downregulating BCL2 expression, while BAX and BAD were upregulated. These findings are inconsistent with previous findings on STZ treated INS-1 cells. On the other hand, treating the INS-1 cells with 2.5 and 5 µM of CA acid reversed the STZ effect and protected against apoptosis [34].

In summary, our data demonstrated that CA protected pancreatic β-cells against STZ-induced damage and reversed all the other effects on insulin content, secretion glucose uptake, MDA levels, and ROS production. The underlying mechanism for this effect may involve multiple mechanisms, through inhibition of mitochondria-mediated apoptosis induction of the PI3k/AKT. PDX-1 signal pathway for insulin production and secretion. Our findings provide new insights on the potential role of CA in protecting pancreatic β-cells against apoptosis.

## 4. Materials and Methods

### 4.1. Culturing of INS-1 Cell Line 

RPMI 1640 medium was used to culture and maintain INS-1 (832/13) cells (a gift from Dr. C. Newgard; Duke University, Durham, NC, USA) as described previously [35,36].

### 4.2. Cell Viability Assessment 

INS-1 (20 × 10^3^ cells) were incubated in the presence of various concentrations of CA (2.5, 5 and 10 µM; dissolved in DMOS) in a 96-well plate for 24 h, then treated with STZ (3 mM) for 3 h [37]. Next, cells were incubated (at 37 °C for 2 h) with 10 µL of (5 mg/mL) MTT solution (Sigma-Aldrich, St. Louis, MO, USA). Dimethylsulfoxide (DMSO) was then used to dissolve formazan crystals, then the absorbance at 570 nm was measured. Cell viability was calculated using the following formula based on the absorbance values: % cell viability = (OD 570 nm of sample/OD 570 nm of control) × 100. As a negative control, untreated cells were employed.

### 4.3. Apoptosis Assay

Cells were treated with various concentrations of CA (2.5, 5, and 10 M) for 24 h before being exposed to STZ (3 mM) for 3 h. Then, cells were resuspended in 400 µL of Annexin-V Binding Buffer (BD Biosciences, Santa Cruz, CA, USA). This was followed by staining in the dark for 10 min using 5 µL of Annexin V-FITC and propidium iodide (PI). The data was analyzed using a flow cytometer (BD FACS Aria III Becton Dickinson, Santa Cruz, CA, USA).

### 4.4. Insulin Secretion Assays

Glucose-stimulated insulin secretion (GSIS) assay was carried out as previously described [35]. In brief, treated cells were washed with SAB (secretion assay buffer) containing 2.8 mM glucose, followed by 2 h of normalization using the same buffer. Subsequently, 1 mL SAB (containing either 2.8 or 16.7 mM glucose) was used to incubate the cells for 1 h. According to the manufacturer protocol, insulin secretion was measured using a rat insulin ELISA kit (Mercodia, Uppsala, Sweden). The total protein extraction was performed using the protein extraction reagent (M-PER) and quantified by Pierce BCA protein assay (Thermo-Fisher Scientific, Rockford, IL, USA). The rat insulin ELISA kit was used (diluted 1:250) to measure the insulin, which was then normalized against the total protein amount.

### 4.5. Measurement of Glucose Uptake 

To evaluate the level of glucose uptake in INS-1 cells, we used a glucose uptake assay kit (Invitrogen #N13195, Carlsbad, CA, USA) according to the manufacturer’s instructions. Briefly, cells were incubated with (2NBDG) for 60 min, followed by washing with cell-based assay buffer, then examined by a FITC flow cytometry detector (Excitation/Emission 485/535 nm).

### 4.6. Reactive Oxygen Species (ROS) Intracellular Measurement 

As instructed by the manufacturer, the hydrogen peroxide (H_2_O_2_) assay was carried out. Briefly, 20 × 10^4^ cells/well were seeded in a 96-well plate in 100 µL medium containing various concentrations of CA. The cells were incubated at 37 °C in a 5% CO_2_ incubator for 3 h after treatment with STZ (3 mM). The H_2_O_2_ substrate solution (20 µL) was added and incubated at 37 °C for 3 h. Afterward, ROS-Glo detection solution (100 µL) was added to each well and set at room temperature for 20 min, with instantaneous luminescence measured using a plate reader. The relative luminescence unit was calculated based on the average results (RLU).

### 4.7. Malondialdehyde (MDA) Assay 

According to the manufacturer’s protocol, the malondialdehyde ELISA kit (#RK09070, ABclonal, Wuhan, China) was used to detect the MDA level. In brief, after 24 h of CA treatment (2.5 µM and 5.0 µM), STZ (3 mM) was added and incubated for 3 h. The cells were then collected by trypsin-free digestion with EDTA and resuspended by adding PBS and vertex thoroughly. The suspension was then centrifuged at 3000 rpm for 10 min at 4 °C, and the supernatant was used to detect the level of MDA.

### 4.8. Immunoblotting Analysis 

Total protein extraction from treated and untreated cells was performed using M-PER (including a protease inhibitor cocktail) followed by gel electrophoresis (SDS-PAGE) before being blotted onto a nitrocellulose membrane (Bio-Rad, Hercules, CA, USA). The membrane was incubated in the blocking buffer (5% skimmed milk in TBST buffer), then treated with primary antibodies, including PDX1 (1:3000, #ab47267, Abcam, Cambridge, UK), BAD (A19595), BAX (A2211), PARP (A19596), AKT (#A17909), BCL-2 (#A19693), Phospho-AKT-S473 (#AP0637), and PI3Kinase p110 (#A19742), and from Abclonal technology (Woburn, MA, USA), Pro/Insulin (#81385), from Cell Signaling Technology (CA, USA) or β-actin (#A5441, Sigma-Aldrich, Hamburg, Germany) antibodies. Finally, the membrane was treated (for one hour at room temperature) with the secondary antibodies (#7076S and #7074S, Cell Signaling Technology, Santa Cruz, CA, USA). Chemiluminescence was detected using a Bio-Rad Enhanced chemiluminescence (ECL) substrate kit (Bio-Rad, Hercules, CA, USA). Protein bands were detected using Bio-Rad Image Lab software (Bio-Rad, Hercules, CA, USA). Image J software was used to calculate the band counts. As an endogenous control in all studies, β-actin was employed.

### 4.9. Statistical Analysis

One-way ANOVA nonparametric tests were used for statistical analysis. GraphPad Prism was used for all statistical analyses (version 8.0.0 for Windows, GraphPad Software, San Diego, CA, USA, www.graphpad.com. The data is presented as a mean ± standard deviation (S.D.). Differences were considered significant at *p* < 0.05. 

## 5. Conclusions

CA possesses antidiabetic properties, enhances glucose uptake, and suppresses ROS and MDA levels in pancreatic β-cells. As a result of our findings, more studies into the potential use of CA in preventing and treating T2D may be pursued. However, it is crucial to point out that the conclusions of this study are based on an in vitro model. Therefore, more in vivo and clinical research are recommended to support our findings. Our findings also suggest that diet composition could be a prevention strategy to preserve pancreatic β-cells function before the disease development.

## Data Availability

Data is contained within the article.
